# A challenge for healthcare system resilience after an earthquake: The crowdedness of a first-aid hospital by non-urgent patients

**DOI:** 10.1371/journal.pone.0249522

**Published:** 2021-04-02

**Authors:** You-Xuan Lin, Chi-Hao Lin, Chih-Hao Lin

**Affiliations:** 1 National Center for Research on Earthquake Engineering, Taipei, Taiwan; 2 Department of Emergency Medicine, National Cheng Kung University Hospital, College of Medicine, National Cheng Kung University, Tainan, Taiwan; University of Hong Kong, HONG KONG

## Abstract

After a violent earthquake, the supply of medical services may fall short of the rising demand, leading to overcrowding in hospitals, and, consequently, a collapse in the healthcare system. This paper takes the emergency care system in Taiwan as the research context, where first-aid hospitals are ranked to three levels, advanced, intermediate, and general, and, currently, emphasizes on a general emergency responsibility hospital. Having limited capacity and capability, a general emergency responsibility hospital treats minor and moderate injuries, from which the majority of earthquake-induced casualties suffer. The purpose of this study is to analyze the impact of this group of earthquake-induced non-urgent patients on the performance of a hospital. A patient flow model was built to represent patients’ paths throughout emergency care. Based on the model, discrete event simulation was applied to simulate patients’ trajectories and states of a hospital under four seismic scenarios, where patient visits are 1.4, 1.6, 1.9, and 2.3 times the normal number. A healthcare performance index, Crowdedness Index (CI), is proposed to measure crowdedness on a daily basis, which is defined as the ratio of the average waiting time for treatment to the recommended maximal waiting time. Results of simulations rendered the establishment of empirical equations, describing the relation between the maximum CIs and the patient growth ratios. In the most severe case in this study, the maximum CI exceeds 92 and it takes 10 days to recover from the quality drop. This highlights the problem a general emergency responsibility hospital may encounter if no emergency response measure is implemented. Findings are provided pertaining to the predication of a recovery curve and the alarming level of patient increase, which are supportive information for preparedness planning as well as response measure formulation to improve resilience.

## Introduction

Hospital crowdedness caused by a surge of mass casualties is one of the major obstacles to healthcare system resilience in the face of seismic disasters. Resilience is an important concept in the area of disaster risk reduction, which concerns the ability of a community to “resist, absorb, accommodate, adapt to, transform and recover from the effects of a hazard in a timely and efficient manner [1].” To put it simply, a resilient community is one that mitigates the impact of a disaster as much as possible and resumes its normal state as quickly as possible. Playing a critical role in mortality minimization and physical recovery of the population, a healthcare system is integral to a resilient community. In the context of seismic disasters, damage to building structures and non-structural components, e.g., ceilings and medical devices, reduces a hospital’s capacity to provide emergency medical services. Even if a hospital survives and maintains its full functionality to treat and accommodate patients, long queues may still be formed by rushing patients and overwhelm the emergency department (ED). The consequential prolonged waiting intensifies patients’ physical and mental sufferings, leading to worse consequences, deterioration and even death. Therefore, post-earthquake crowdedness in emergency healthcare facilities is an essential problem to be tackled. Thanks to the well-developed methodology of earthquake loss estimation, specialized modules in software tools, like HAZUS [2] and TELES [3], have made the casualty estimate of any seismic event feasible. Still, a gap lies between the information about the estimated medical demand and the prediction of the likely induced impact on emergency care institutions while the latter is a practical problem related to earthquake preparedness planning and response measure formulation. To make up the gap, in this paper, we take the emergency healthcare system in Taiwan as the research context and demonstrate the relations between patient number growth ratios and emergency care performance decline as well as recovery across days after earthquakes by two performance measures, Crowdedness Index and Quality.

Supply-demand imbalance has long been a problem that bothers emergency healthcare facilities worldwide. Researchers have been attempting to address the queuing problems in hospitals with underlying causes identified and plausible solutions raised [4]. Asplin et al. [5] proposed a conceptual model of ED crowding that decomposes the ED operation to three parts, input, throughput, and output, and listed relevant factors of each part to ED crowding. Input, or patient visits, is the part that a hospital has less control over. A study based on an ED in Canada pointed out that ambulatory or non-urgent patients who should have visited a walk-in clinic instead of an ED are the main culprit for the long average patient length of stay [6]. With increased patient volumes but limited resources, researchers resorted to staffs’ workload adjustments [6], optimal staff scheduling [7–9], and efficient resource utilization regarding spaces [10] or devices to make the best use of available resources with no or the least additional costs incurred. Some effort was made to develop supportive tools which utilize records in the ED information system to predict demand in the following few hours and help administrators manage anticipated trouble in advance [11], or to evaluate efficiency of various strategies, supporting administrators to improve ED performance [12].

Due to the unpredictable nature, widespread devastation, and a large population involved, the post-earthquake performance of emergency medicine is a more complex problem to be managed. Hassan and Mahmoud [13] modelled a healthcare system as a network where interdependence holds among a cluster of healthcare facilities and crucial infrastructures in a community (e.g., lifelines and transportation). Approaches of disaster preparedness assessment of hospitals were investigated and developed to help detect weakness and improve preparedness for unexpected disaster outbreaks [14, 15]. Other studies narrowed down their research focuses and emphasized on the failure analyses within hospitals, analysing functionality loss either in one single healthcare facility or multiple facilities in a region. This type of study involves dynamic modelling of patient arrivals and trajectories, fragility study of hospital structures and non-structural components, and examination of the efficiency of different repair strategies considering time and money invested in (e.g., [16–19] for one single hospital; [20] for multiple hospitals).

Though previous studies have covered an extensive range from ED functionality inspection, management and policies in response to disasters, preparedness assessment, to comprehensive perspectives that involve multiple healthcare facilities and relevant infrastructures, a fundamental question is not yet answered: what the relation between the patient inflow volumes and the emergency care capacity is. This is an essential point to be considered when it comes to the decision-making about the initiation of emergency response and coordination among medical facilities in a region. As a strategy to ease the pressure of particular well-known large hospitals, patient diversion based on acuity levels is believed effective, especially by removing non-emergency patients [21]. However, if this strategy is to be put into strict practice, the influence of the mass non-urgent patients on hospitals assigned to take over remains uncertain. Take two recent large-scale earthquakes in Taiwan for example. In the Hualien Earthquake (peak ground acceleration (PGA) > 400 gal) occurring on February 6, 2018 in Hualien (eastern Taiwan), over 90% of patients fall in the category of lower acuity levels (AL3 –AL5) [22]. Similarly, in the Kaohsiung Earthquake (southern Taiwan) with PGA over 400 gal occurring on February 6, 2016, 87% are intermediate to mild injuries [23]. Therefore, this paper aims to address two main research questions with focuses exclusively on the impact of the post-earthquake non-urgent patient rush on a hospital in charge of non-urgent injuries: (1) Confronted by different volumes of non-urgent patients, how would the recovery curve of emergency quality be like? (2) How can the non-urgent patient number growth raise the alarm about the upcoming collapse of emergency care and serve as a sign to start emergency response?

Researchers conducted discrete event simulation (DES) to simulate the operation of emergency care in a hospital and capture queuing behaviors. Patients’ traces over the course of a visit in a hospital for emergency care were modelled as a series of events (or medical services). Queuing between events were manipulated by setting finite numbers of resources for each event and specifying service time distributions. This study is based on an assumption that the structure and non-structure components are not damaged by earthquakes and the resource number keeps constant. Therefore, the only variable that influences hospital performance is the patient number. Scenarios of patients 1.4, 1.6, 1.9, and 2.3 times the normal number were carried out.

As to capture the changing performance of a hospital along with the increasing emergency care demand, a performance measure that quantifies the overcrowding state is proposed in this paper, i.e., Crowdedness Index (CI henceforth). CI is obtained from two temporal values: the average waiting time for treatment of patients in the same triage category by the day and the corresponding advised maximal time for that category. It serves as an indicator of an institution’s ability to provide timely treatment for patients of different acuity levels. While different ways were also used to quantify the extent of overcrowding, for example, the ratio of current ED loading to the overall ED capacity [24], and inpatient bed availability [25], after a disastrous event, an index that reflects the capacity to offer effective and timely treatment provides more relevant information in a life-or-death situation. The other index used in this paper, Quality, takes the inverse of CI and is upper bounded to 1, shedding light on the remaining emergency care quality after seismic impact.

This study provides insightful findings about the emergency care performance under circumstances of earthquake-induced patient surges. The estimated maximum CI and the length of recovery period of emergency care quality allow the medical institutions, decision-makers, relevant practitioners to prepare for the predicament and to take quick and rational actions on the spot. Overall, it is expected to help with pre-disaster preparedness planning for the worst state and post-disaster emergency response measure formulation to suppress the impact of outgrowing patients so that medical institutions can provide the whole community with quality medical services and facilitate disaster resilience of a community.

## Emergency care system in Taiwan

Emergency departments in Taiwan adopt the five-level triage system. Upon arrival, patients are triaged based on acuity levels (AL) of injuries, with level 1 as the most severe and level 5 the least. Each level has the corresponding maximal waiting time for treatment which is to be followed to prioritize more emergency patients. AL1 requires immediate resuscitation, AL2 requires treatment within 10 minutes, AL3 within 30 minutes, AL 4 within 60 minutes, and AL5 within 120 minutes. In this paper, acuity levels (ALs) and triage categories are used interchangeably for the sake of readability. In addition to ranking patients by acuity levels, Ministry of Health and Welfare also ranks first-aid hospitals to one of the three levels based on their capacity and capability to provide emergency care: advanced emergency responsibility hospital, intermediate emergency responsibility hospital, and general emergency responsibility hospital. First-aid hospitals of the lowest rank are not required to have critical care capability. Their primary tasks are oriented to less severely injured patients, or AL3 –AL5. General emergency responsibility hospital is the target being modelled in this study.

## Model of patient flow and parameter setting

### Establishment of patient flow model

Discrete event simulation (DES) was conducted with the help of a Python library, SimPy [26] to simulate the patient flow in the context of emergency care. SimPy enables the automatic generation of patients at designated rates, distributes patients to available resources, and moves patients forward through the assigned flow path. The path patients take and the time they spend on each service are governed by probability distributions, which will be elaborated below.

**Fig 1:**
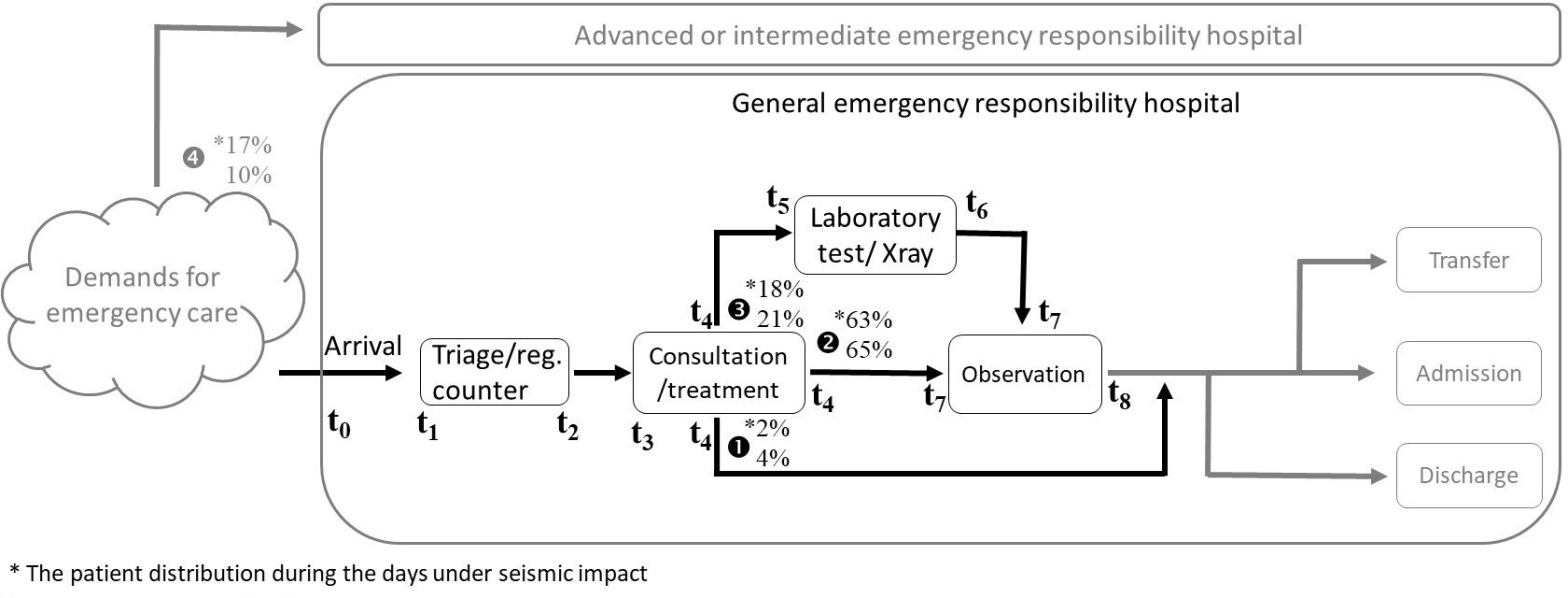
Patient flow of first-aid hospitals.

Based on Favier et al. [16] and Côté [10] as well as some modifications made to approximate the emergency care procedure in Taiwan, the patient flow paths throughout first-aid hospitals were modelled, as shown in Fig 1.

The patient flow was designed to split into two separate routes, one for urgent patients (AL1 and AL2) and the other for less acute patients (AL3, AL4, and AL5). As generated, patients will be distributed by the assigned proportion to one of the two routes that represent first-aid hospitals of different ranks. In this paper, we exclusively focus on the queuing behavior of minorly/moderately injured patients and assume that they are all ushered to a general emergency responsibility hospital. Events happening in the route for urgent patients in advanced or intermediate emergency responsibility hospitals will not be further described and analyzed here.

As entering the modelled general emergency responsibility hospital, patients take one of the three paths, which is randomly determined against a distribution (the percentage marked on Fig 1). Overall, when one patient arrives, he/she would be first triaged and then wait for the doctor’s consultation and treatment. Once the treatment finishes, the forthcoming path diverges as follows: (1) leaving the institution immediately after treatment, (2) staying under observation, or (3) receiving further examination and being kept for a while for observation. The procedure of emergency medical care would terminate by one of the three output options, admission in the current hospital, transfer to other institutions, or discharge (to rest at home). While the output mechanism was already mapped in our model, for the ease of the current discussion on the relation between input and throughput phases, at present, we disregard patient output as a concern for the performance of a first-aid hospital.

### Patient distribution and path assignment

Based on real data and estimation, Favier et al. [16] proposed the percentage of each triage category and the probability of each category being assigned to one of three paths. This study primarily adopted their figures, but some changes were made. Two major changes were: (1) the percentage of AL1&AL2 patients, and (2) the path taken by AL5 patients.

First, considering the fact that large-scale earthquakes may increase the cases of deadly injuries, we calibrated the percentage of patient categories in Favier et al. by using real data to differentiate the percentage on normal days and under seismic scenarios. Two sets of data were exploited for this purpose: (1) the month-long patient statistics of hospitals in Tainan Metropolitan Region (southern Taiwan), by which AL1&AL2 patients take up 10% [27], and (2) patient data collected from a hospital in 2018 Hualien Earthquake, by which AL1&AL2 patients constitute 17%. By aligning the percentages of urgent patients (AL1&AL2) in Favier et al. with those in the real data (10% on normal days and 17% during the period of seismic impact), we derived two sets of patient distributions for two conditions, as shown in Table 1.

**Table 1:** Distribution of patient categories.

	AL3	AL4	AL5	AL1+AL2
Normal	52%	32%	6%	10%
Seismic	50%	30%	3%	17%

Second, in Favier et al, a proportion of AL5 patients would leave directly from triage without seeing the doctor at all. In our model, this alternative was avoided since this seems unacceptable in Taiwan. Therefore, AL5 patients were reallocated to Path 1 and 2. The revised distribution of paths by acuity categories are presented in Table 2.

**Table 2:** Distribution of patients to paths by acuity levels.

	AL3	AL4	AL5	AL1+AL2
Path1	0%	0%	75%	0%
Path2	76%	76%	25%	0%
Path3	24%	24%	0%	0%
Path4	0%	0%	0%	100%
	100%	100%	100%	100%

With the distribution of patient categories and the path distribution for each category, the parameter that determines the path of each patient upon generated in our model was obtained (as shown in Table 3). Patients taking Path1 are composed of 75% AL5 patients, which is 4% on normal days and 2% on the seismic condition of all arriving patients. Path2 is a path for 65% patients on normal days and 63% patients after an earthquake, which are made up of 76% AL3 and AL4 patients as well as a quarter portion of AL5 patients. 21% and 18% of all patients would be assigned to Path3, which covers 24% AL3 and AL4 patients. Finally, Path4 is a separate route for urgent patients (i.e., AL1&AL2 patients) leading to advanced or intermediate emergency responsibility hospitals. 10 and 17 out of 100 patients would all be directed to Path4.

**Table 3:** Distribution of arriving patients to paths.

	Path1	Path2	Path3	Path4
Normal	4%	65%	21%	10%
Seismic	2%	63%	18%	17%

### Patient arrival and resource occupancy

After an earthquake, patients arrive at an emergency care facility in a pattern different from normal ways. Favier et al. [16] considered both regular patient visits and seismic casualties, and their dispersion over time to estimate patient arrival rates within four days after earthquakes, which will be referred to as the busy phase hereafter. This study adapted the estimated arrival rates to generate patients over time.

**Fig 2:**
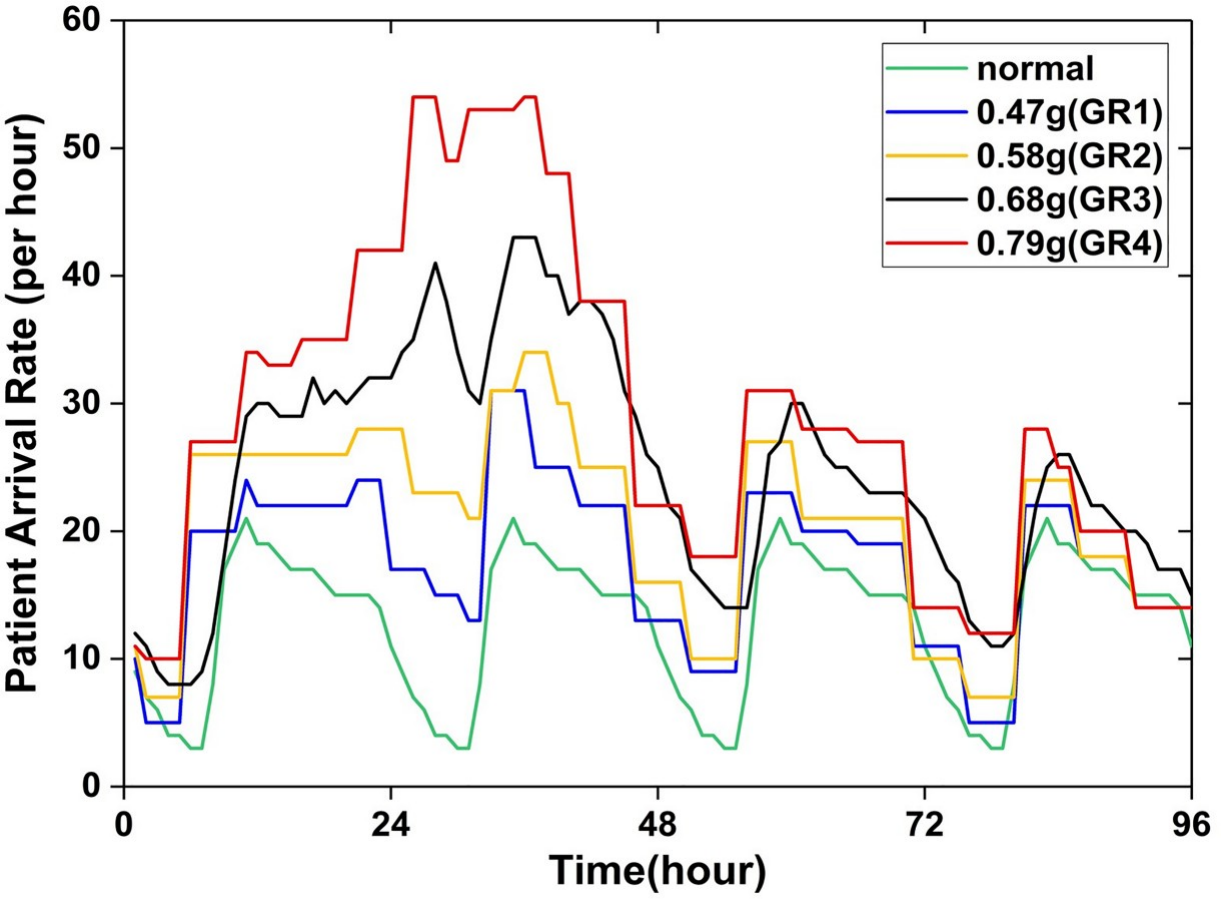
Patient arrival rates within the four-day busy phase and on normal days (adapted from Favier et al. [16]).

As displayed in Fig 2, instead of being fixed or smoothed, the arrival rates fluctuate with the passage of time during the busy phase. Arrival rates in five scenarios in Favier et al. were adapted for simulation in this study, one in normal situation and four after earthquakes. Since the casualty number varies in different seismic events even with the same intensity, the seismic intensity is not an appropriate variable for this study. Instead, the ratio of the patient visit volume during the busy phase to that on the normal condition, or growth ratio for short, is more reliable and also adaptable for flexible interpretation and generalization. The four seismic scenarios are then named orderly as GR1 (growth ratio = 1.4), GR2 (growth ratio = 1.6), GR3 (growth ratio = 1.9), and GR4 (growth ratio = 2.3), with GR1 having the lowest growth ratio and GR4 the highest.

As one patient is generated in the simulation program, he/she will be moved forward along the assigned path to receive medical services and, meanwhile, occupy one resource unit of the particular service. One resource unit here refers to a combination of all labours and devices needed to serve one patient, instead of denoting to any particular staff or items. That is, the resource number of each service represents the upper limit of the patient number accepted for the same service at the same time. The length of time spent on each medical service and the gross resource number are two parameters to be set in the program. The time distribution of each service and the resource number are based on previous studies with some adjustments made after initial testing, as presented on Tables 4 and 5. It is supposed that certain probability distributions govern the length of service time. The time each medical service takes is determined by random sampling from the corresponding distribution. The resource number of Observation is set to be infinite in that the space for patients to rest can be any vacant space, such as benches in the hall.

**Table 4:** Probability distribution of service time.

Service	Probability distribution	Source
Triage/registration	Gamma(4.5, 0.7)	Favier et al. [16]
Consultation/treatment	Tri(15, 45, 90)	Favier et al. [16]
Observation	Tri(0, 15, 60)	Adapted from Favier et al. [16]
Lab/X-ray	Tri(30, 75, 120)	Favier et al. [16]

Parameters of probability distribution are set in minutes.

**Table 5:** Resource number of each service.

Service	Quantity	Source
Triage/registration	1	Favier et al. [16]
Consultation/treatment	13	Favier et al. [16]
Lab /Xray	6	Obtained by testing
Observation	∞	Obtained by testing

### Model validation

Overall, the simulation in each scenario was repeated for 300 times to level out the effects of outliers due to sampling. The length of a run varies from 12 to 20 days depending on the time needed to recover from the seismic effects. The first six days are kept constant for all scenarios, with the leading two days as the pre-earthquake situation followed by the four-days busy phase during which patients injured in the earthquake rush in. The remaining days vary in different scenarios in order to completely exhibit the recovery curve, for some scenarios require longer recovery time.

The simulation model with a set of parameters was tested to ensure that it could by and large represent the operation of emergency care institutions. To confirm that the resource number and service time of each medical service were reasonably set, we compared the simulation results with the real census data (Table 6). In our model, every patient (AL3 –AL5) waits for approximately 9 minutes before receiving treatment on normal days. As for the real situation, according a report surveying all EDs in Taiwan [28], AL3, AL4, and AL5 patients’ waiting time for treatment is on average around 9 to 10 minutes, as shown in Table 7. While the hospital levels used in that report are from a different ranking system that mainly evaluates the general capacity of a hospital, including its size, staff, capability, and missions, general emergency responsibility hospitals can be compared to district hospitals. In comparison, the simulation result (9 minutes) is very close to the real data (9 to 10 minutes). These figures suggest that our model is generally able to, if not accurately, observe the normality of a general emergency responsibility hospital in reality.

**Table 6:** Comparing average waiting time for treatment from simulation and real data [28].

	AL3	AL4	AL5
Simulation result	9.0	9.0	9.0
Real data (district hospital)	10.0	8.9	9.4

**Table 7:** Average waiting time for treatment by ALs and hospital levels in 2012 in Taiwan [28].

	AL1	AL2	AL3	AL4	AL5
Medical center	6.1	10.0	11.9	12.4	12.6
Regional hospital	7.6	7.3	8.2	10.5	11.4
District hospital	5.8	8.0	10.0	8.9	9.4
Average	6.6	7.9	9.5	9.9	10.6

## Crowdedness index

Crowdedness Index (CI) is a measure that evaluates a first-aid hospital by its ability to provide timely treatment for patients of different ALs. Ministry of Health and Welfare in Taiwan has issued a guidance on triage, in which the maximum waiting time for each ALs is advised. By exploiting the guidance, CI is then defined as the quotient of average patient waiting time for treatment over the advised maximum waiting time grouped by ALs. Therefore, each level has its own CI calculated. The calculation to obtain CI is described below in Eq 1, Eq 2 and Eq 3:

$${CI}_{AL3,j}=\frac{\sum_{p=1}^{N_{AL3,j}}\left(t_{3}^{p}-t_{0}^{p}\right)}{N_{AL3,j}∙T_{AL3}}$$ (1)

$${CI}_{AL4,j}=\frac{\sum_{q=1}^{N_{AL4,j}}\left(t_{3}^{q}-t_{0}^{q}\right)}{N_{AL4,j}∙T_{AL4}}$$ (2)

$${CI}_{AL5,j}=\frac{\sum_{r=1}^{N_{AL5,j}}\left(t_{3}^{r}-t_{0}^{r}\right)}{N_{AL5,j}∙T_{AL5}}$$ (3)

where *p*, *q*, and *r* stand for every single AL3, AL4 and AL5 patient arriving on the *j* day and receiving treatment, *N*_*AL3/4/5*_ is the total number of *p*, *q*, and *r*, and *T*_*AL3/4/5*_ is the advised maximum waiting time, 30 minutes for AL3, 60 minutes for AL4, and 120 minutes for AL5.

The period of time in the hospital that CI concerns is from arrival to treatment, or the time interval between *t*_*3*_ and *t*_*0*_, as marked in Fig 1. It should be noted that the term “waiting time” may be used differently in studies of different research purposes. It was defined as the time difference between triage and treatment (or consultation) in a study searching for the best solution of emergency physician scheduling [8]; in another study, it was used to refer to the time interval from arrival to departure as the target of machine learning-based prediction [29]. In the current study, we are concerned about how long it takes for a patient to reach a doctor upon arrival, the waiting time here is then defined as the time interval from arrival to treatment which includes the time expended on queuing for triage, triage, and queuing for consultation, also known as door-to-doctor time (DTDT) [30]. Optimally, we would expect the CI to be equal or below 1 so that patients can be treated within the maximum waiting time and any life-threatening condition could be at best controlled. Oppositely, if the CI exceeds 1, it means that an emergency care institution fails to provide timely treatment and patients are at risk of ineffective treatment and adverse health conditions.

## Results

### Crowdedness Index in different scenarios

**Fig 3:**
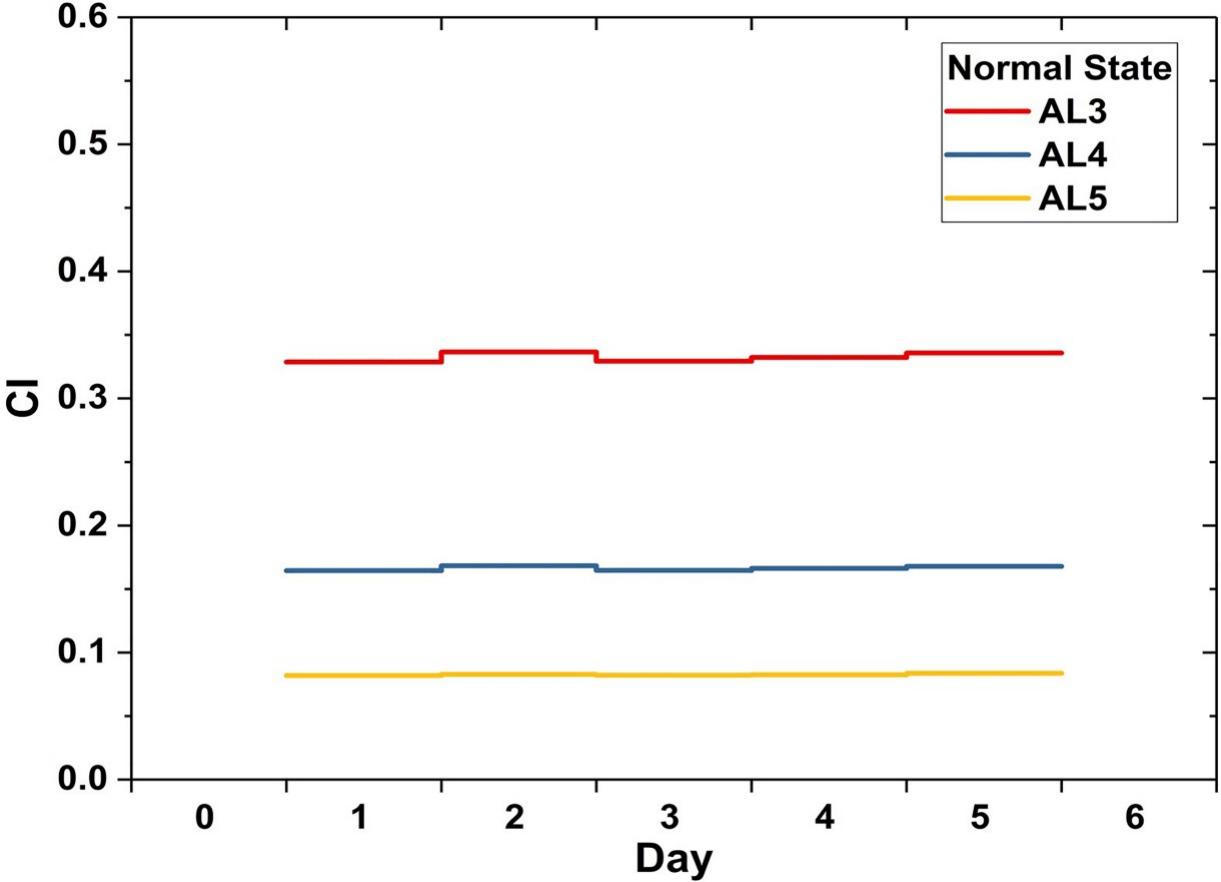
Crowdedness Index in the normal situation.

Crowdedness Index is a measure of an institution’s capacity to provide timely medical services. Patients of different acuity levels are tolerant of different lengths of waiting time, as elaborated in the previous chapter. A proper emergency care institution is supposed to be able to manage the ordinary demands and supply medical emergency treatment within the tolerable waiting time. For the modelled general emergency responsibility hospital in this study, the CIs of the three levels in the normal situation are all below 0.5, indicating that on average less than half the advised maximum waiting time is needed for patients to receive treatment. As shown in Fig 3, the CIs and the injury severity have positive correlation. The average CI is 0.33 for AL3 patients, 0.17 for AL4 patients, and 0.08 for AL5 patients. The positive correlation is a result of patients competing for the same set of resources while the higher-level patients are actually less tolerant of waiting.

**Fig 4:**
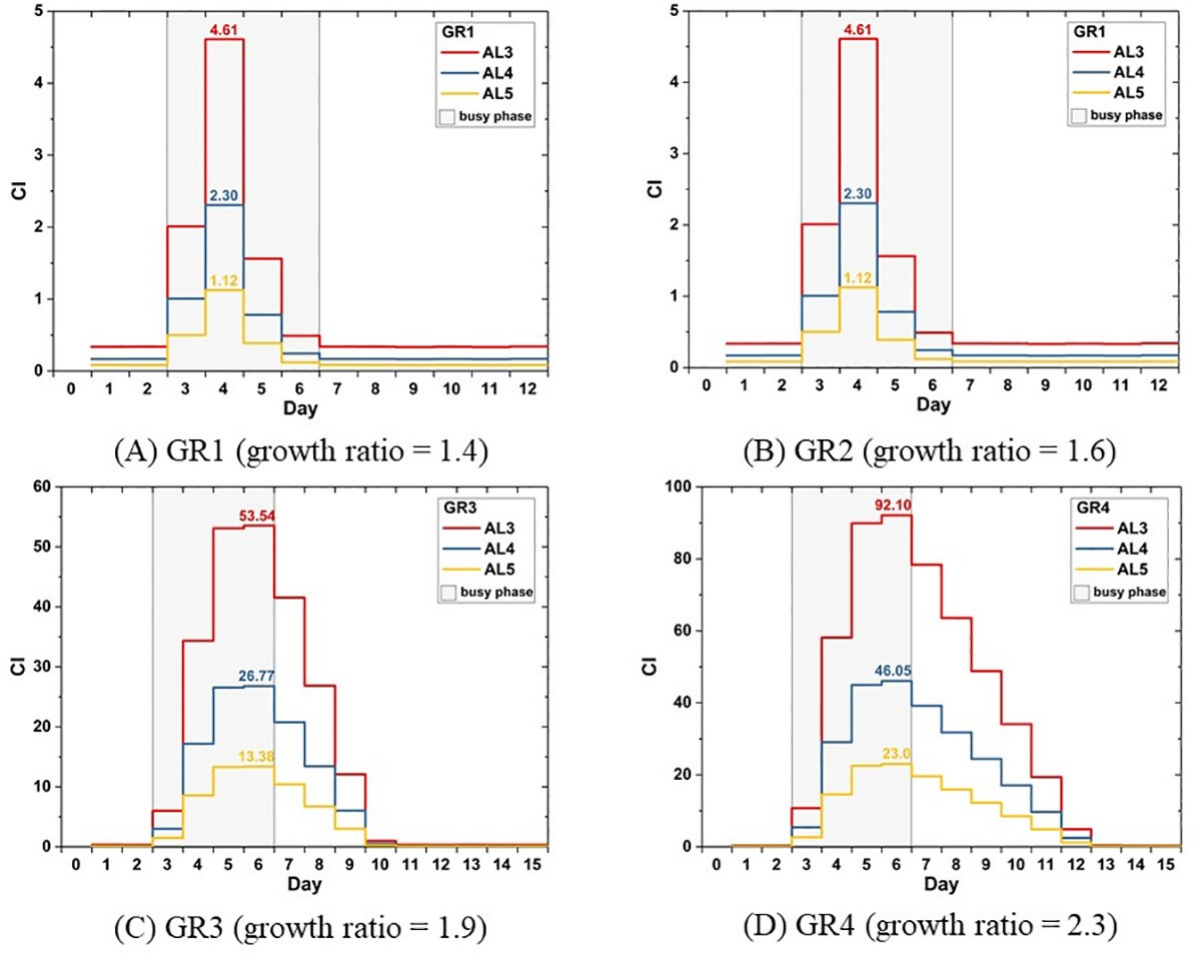
Crowdedness Index under the influence of earthquakes.

Fig 4 exhibits the rise and fall of CIs under the influences of different patient volumes. For the sake of clarity, the four plots (Fig 4A–4D) for different scenarios are on different scales of day and CI. The shade signifies the four-day busy phase during which earthquake-induced casualties visit the hospital. When the patient number increases by 1.4 times during the busy phase, the maximum CIs will be 4.61 for AL3, 2.30 for AL4, and 1.12 for AL5 (Fig 4A). With the patient visit volume 1.6 times the volume in normal state, the maximum CIs rise to 22.09 for AL3, 11.05 for AL4, and 5.53 for AL5 (Fig 4B). With an increase by almost twice the number in ordinary days, the longest waiting time for AL3, AL4, and AL5 patients will respectively be 53.54, 26.77, 13.88 times as long as the advised (Fig 4C). Finally, the most intensive scenario in this study, where the growth ratio of the patient number in the busy phase is 2.3, results in the waiting time 92.10, 46.05, and 23.0 times the length of the advised (Fig 4D).

In this section, we have highlighted the maximum values of CIs in different scenarios and exhibited an overview of changing CIs by days. The maximum CI indicates the worst state subjected to different casualty sizes. While CI offers us information about the emergency care performance on a given day, using different ways to analyze and interpret the data of CI helps obtain information for practical purposes. In the following sections, two ways of analyses are provided. First, the maximum CI is further analyzed in relation with growth ratios to reach a set of empirical equations that can be adopted to estimate states of a hospital in other scenarios. Second, the CIs are viewed from a different perspective to present the decline and recovery of emergency care quality. In this way, the concept of resilience can be brought in.

### The relation between the patient number and maximum CI

**Fig 5:**
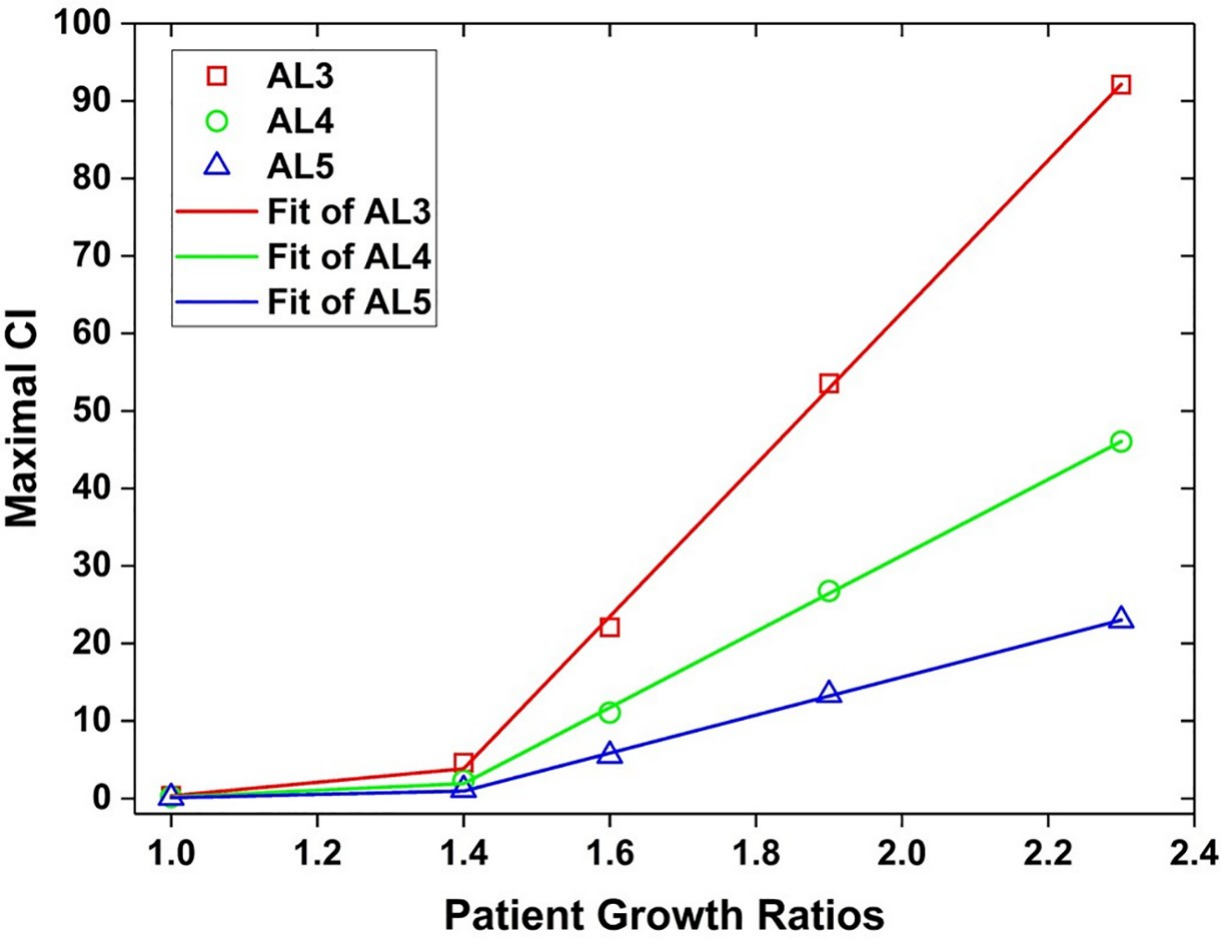
The patient growth ratios and effects on CI.

With the patient arrival rates (see Fig 2) assigned for the five scenarios, patients are increased by a factor of 1.4, 1.6, 1.9, and 2.3 respectively during the four-day busy phase. It was found that the patient growth ratios impose certain effects on the maximum value of CI in each AL group. Based on the simulation results, we worked out three equations that describe the relation between the two variables, patient growth ratio (*P*) and the maximum *CI*_*AL*_. Graphical presentation of the relations is shown in Fig 5.

$$Max{CI}_{AL3}=\left\{ \begin{array}{cc} -8.42+8.75P & P\leq 1.4\\ -133.56+98.13P & P>1.4 \end{array}\right.$$ (4)

$$Max{CI}_{AL4}=\left\{ \begin{array}{cc} -4.19+4.36P & P\leq 1.4\\ -66.78+49.07P & P>1.4 \end{array}\right.$$ (5)

$$Max{CI}_{AL5}=\left\{ \begin{array}{cc} -2.08++2.16P & P\leq 1.4\\ -33.4+24.53P & P>1.4 \end{array}\right.$$ (6)

Apparently, the AL3 group is the most vulnerable one subjected to the increase of patient numbers because of its least tolerant quality among the three groups. By the derived equation (Eq 4), when the AL3 patient number is 1.5 times the regular number, it is predicted that the maximum CI_AL3_ will exceed 10. When the patient number increases about twofold, the maximum CI_AL3_ rockets up to 62, which means that for an AL3 patient to approach to the doctor, it will take a time 62 times longer than the optimal one (30 minutes). Even for AL5 patients, who are triaged as the least urgent patients and allow longer waiting time, the maximum CI_AL5_ is almost as great as 16 due to the doubled patient number.

In conclusion, we can find that maximum CI has a drastic reaction to the growth of patient numbers especially when the growth ratio is beyond 1.4. Even though the excessively lengthy waiting time may not necessarily be fatal yet for those patients, it cannot be neglected that their suffering, both mental and physical suffering, is prolonged and intensified during the process. Consequently, it will make the already tough recovery period, regarding both personal lives and the whole community, even more challenging.

### Emergency care quality

The CI data were analyzed from another perspective to generate a straightforward representation of the emergency care quality. Eq 7 presents how to achieve emergency care quality (*Q*_*i*_) by using *CI*_*i*_, where *i* stands for AL3, AL4, AL5. In brief, Q is determined by taking the minimal value between 1 and the reciprocal of CI. A properly functional general emergency responsibility hospital has its Q to be 1 where patients can be treated in time. Oppositely, any value lower than 1 notifies overburden and a decline in its quality. Inheriting the property of CI, Q also has its focus on a hospital’s ability of timely and effective healthcare provision.

$$Q_{i}=min\left(\frac{1}{{CI}_{i}},\;1\right)$$ (7)

According to the simulation results, CIs on normal days, regardless of acuity levels, are all far lower than 1 (see Fig 3). That is, ordinary patient visit volumes are perfectly within its capacity and the emergency healthcare quality is in its proper condition, i.e., Q = 1 as defined above. In seismic scenarios, while the CIs soar rapidly in all of the four cases, on the first day of the busy phase in the GR1 scenario, the hospital remains its proper quality with CI_AL3_ below 1 (Fig 4A). This suggests that an increase in CI is not necessarily equivalent to a significant decline in the emergency care quality.

**Fig 6:**
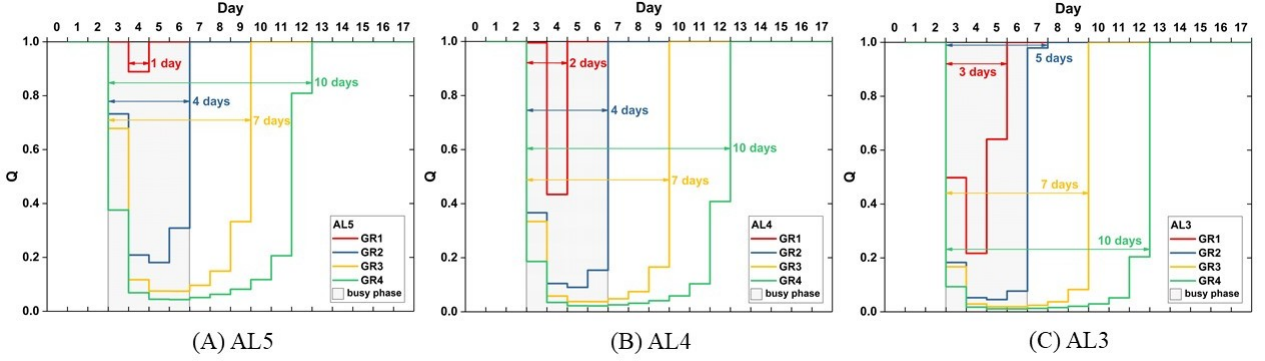
Emergency care quality drop and restoration.

Alternatively, Q is efficient in capturing the drop and restoration of the emergency care quality, as plotted in Fig 6. Fig 6 illustrates the impact of increased patient volumes on the emergency care quality respectively for the three patient categories. It can be observed that Q slumps immediately on the first day of busy phase, except for AL5 in GR1 scenario where the Q remains 1 and the drop delays by one day (see Fig 6A).

As to recovery, the process of quality drop and restoration, the period is prolonged as patient volumes increase. As expected, GR4 results in the longest recovery time and the GR1 the shortest. However, when compared across acuity levels, the quality recovery period for more acute injuries is not necessarily prolonged. No difference in the length of recovery period was observed across levels in GR3 and GR4 scenarios. 7 days and 10 days are required respectively for all patient categories. However, it takes additional days in GR1 and GR2 to recover the quality of AL3. In conclusion, this may suggest that when the patient number inflates to a certain extent, the quality loss caused by queueing has equal impacts on patients of all acuity levels in terms of recovery.

## Discussion

In summary, this study built a model with a set of parameters to simulate patient flow in a general emergency responsibility hospital. By repeating simulations with different patient arrival rates, time records of every patient entering and leaving each medical service were acquired. After analyzing the data, results regarding changes of CI across time, the relation between the maximum CI and the patient growth ratios, and the Quality were attained. Specifically, these results help answer the two questions significant for pre-disaster preparedness planning and post-earthquake emergency response: (1) Confronted by different volumes of non-urgent patients, how would the recovery curve of emergency care quality be like? (2) How can the non-urgent patient number growth raise the alarm about the upcoming collapse of emergency care and serve as a sign to start emergency response?

**Fig 7:**
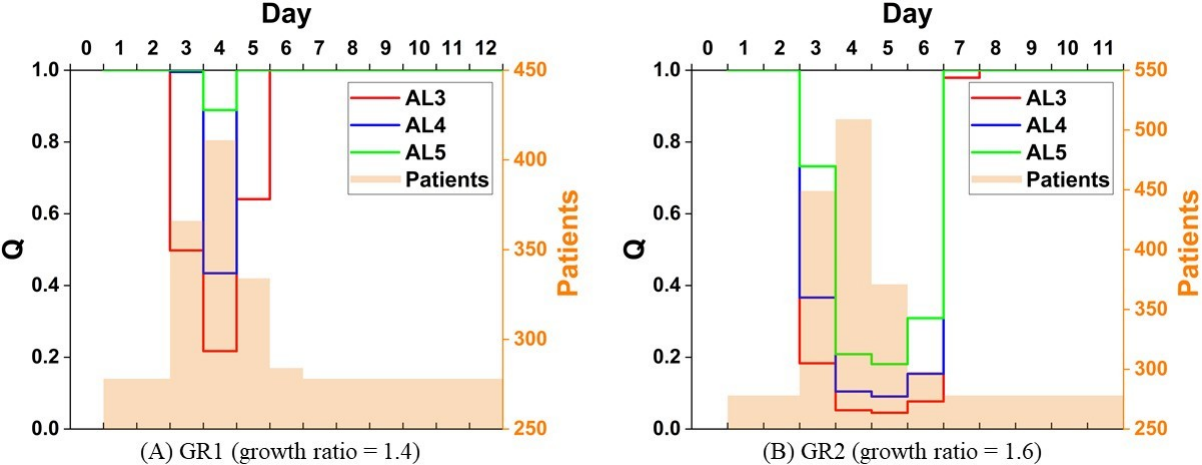
The Quality recovery curve and the daily patient volume.

To answer the first research question, three attributes are relevant in the shape of a recovery curve, which are width, depth, and trough. First, the width of a recovery curve refers to the length of the complete recovery process. As what have been shown above, it takes as long as 7 days and 10 days to retrieve the standard emergency care quality for all triage categories when the patient number grows by a factor of 1.9 and 2.3. When the growth ratio is 1.4 and 1.6, the standard quality can be regained by the time the busy phase terminates, except for AL3 in the latter scenario where one more day is required. As for the depth of the curve, it is the maximum Quality loss that an event would cause. Since the reciprocal of CI serves Quality, the empirical equations of the maximum CI can be applied to obtain the depth of a recovery curve. Finally, the trough of a recovery curve indicates the point where Quality starts to rebound. If we look closely at the results in Quality, aside from the scenario of the mildest patient number increase in our simulation, i.e., GR1, there is a delay by one or two days in the time Quality bottoms out after the patient arrival spikes. The delay is a result of patients stuck in queues and yet to be resolved. When patient volumes surpass a threshold, which might be somewhere between the patient volume between GR1 and GR2 for the modelled general emergency responsibility hospital in this study, the impact of patient influx lingers. Given these findings, a general picture of the recovery curve under a seismic condition, provided that the casualty estimate is available, can be formed regarding its could-be width, depth, and trough. Fig 7 displays the shape of the recovery curve in the scenario of GR1 and GR2 to demonstrate the abovementioned findings. The Quality hits bottom as soon as the patient number reaches the peak in Fig 7A, whereas in Fig 7B it is one day after the patient peak that the Quality hits bottom. The maximum of the daily patient number and the minimum of the Quality are highlighted in bold in Table 8.

**Table 8:** Quality and the patient number in GR1 and GR2.

P = 1.4 (GR1)	Quality (Q)			
Day	AL3	AL4	AL5	Patients
1	1	1	1	278
2	1	1	1	278
3	0.49772	0.99544	1	366
4	**0.21703**	**0.43407**	**0.88892**	**411**
5	0.64057	1	1	334
6	1	1	1	284
7	1	1	1	278
8	1	1	1	278
9	1	1	1	278
10	1	1	1	278
11	1	1	1	278
12	1	1	1	278
P = 1.6 (GR2)	Quality (Q)			
Day	AL3	AL4	AL5	Patients
1	1	1	1	278
2	1	1	1	278
3	0.18319	0.36638	0.73228	449
4	0.05225	0.1045	0.20864	**509**
5	**0.04526**	**0.09052**	**0.18087**	371
6	0.07692	0.15383	0.30889	298
7	0.97913	1	1	278
8	1	1	1	278
9	1	1	1	278
10	1	1	1	278
11	1	1	1	278
12	1	1	1	278

The answer to the second research question can be inferred from the relation between the patient growth ratio and the maximum CI. As demonstrated in Fig 5, the maximum CI increases with the patient growth ratio along a smooth slope when the ratio is below 1.4, beyond which the slope becomes steep. That is to say, 1.4 is the critical figure of the growth ratio that alerts decision-makers. If an earthquake strikes and the estimated emergency healthcare demand is 1.4 times as great as the normal demand, actions should be taken in response to the prospective overcrowding and to prevent medical collapse from happening as a result of the excessive demand. Otherwise, the situations observed in the simulations of this study may be the consequence, including a failure to restore the proper emergency care quality even after the seismic casualty stops visiting, and a delayed rebound in quality by one or two days from the patient visit peak.

## Conclusion

This study is a preliminary attempt to analyse the post-earthquake congestion of healthcare institutions with its current research target on one single general emergency responsibility hospital. In the current study, we have put our exclusive attention on the impact of a sudden increase in patient visits made up of seismic casualties. By means of discrete even simulation, changes in the performance of a hospital faced with different volumes of patient influx were captured and presented by performance measures, CI and Quality. Afterwards, in the discussion of the simulation results, findings regarding the prediction of the emergency care quality recovery curve, and the amount of patient increase as a warning sign forecasting a collapse of the medical supply were reported.

For future work, effort will be taken to model all levels of first-aid hospitals and explore various problems that might influence the proper function of emergency care after an earthquake strikes. The currently established model will be adjusted to represent advanced and intermediate emergency responsibility hospitals with parameters and patient distributions fine-tuned so that we are able to build a virtual emergency care system. By exploiting earthquake loss estimation systems, like TELES for Taiwan [3], to obtain the casualty estimate, the simulation can be conducted on a regional basis and the regional capacity in a real-world context can be examined [31]. On the other hand, while current study kept the resource number constant, the effects of resource increase after the implementation of emergency response measures and the impact of the resource decrease as a result of seismic loss (e.g., power supply system failure [32, 33]) are topics worth further elucidation.

## Supporting information

S1 File (DOCX)
