# Supplementary material for: A challenge for healthcare system resilience after an earthquake: The crowdedness of a first-aid hospital by non-urgent patients
Source: PLoS One. 2021 Apr 2;16(4):e0249522. doi: 10.1371/journal.pone.0249522 (PMC8018621; doi:10.1371/journal.pone.0249522)
Supplement: S1 File — (DOCX) [file pone.0249522.s001.docx]

Fig. 3

| normal | CI | | |
| --- | --- | --- | --- |
| Day | AL3 | AL4 | AL5 |
| 1 | 0.32868 | 0.16434 | 0.08184 |
| 2 | 0.33646 | 0.16823 | 0.08265 |
| 3 | 0.32921 | 0.16461 | 0.08212 |
| 4 | 0.33223 | 0.16612 | 0.0824 |
| 5 | 0.33571 | 0.16786 | 0.08363 |

Fig. 4A

| P=1.4 (GR1) | CI | | |
| --- | --- | --- | --- |
| Day | AL3 | AL4 | AL5 |
| 1 | 0.33691 | 0.16845 | 0.08427 |
| 2 | 0.33798 | 0.16899 | 0.08355 |
| 3 | 2.00917 | 1.00458 | 0.50035 |
| 4 | 4.60756 | 2.30378 | 1.12496 |
| 5 | 1.5611 | 0.78055 | 0.38794 |
| 6 | 0.49004 | 0.24502 | 0.12072 |
| 7 | 0.3397 | 0.16985 | 0.08556 |
| 8 | 0.33806 | 0.16903 | 0.08537 |
| 9 | 0.33179 | 0.16589 | 0.083 |
| 10 | 0.3364 | 0.1682 | 0.08492 |
| 11 | 0.33189 | 0.16595 | 0.0833 |
| 12 | 0.34192 | 0.17096 | 0.08459 |

Fig. 4B

| P=1.6 (GR2) | CI | | |
| --- | --- | --- | --- |
| Day | AL3 | AL4 | AL5 |
| 1 | 0.32888 | 0.16444 | 0.08316 |
| 2 | 0.33906 | 0.16953 | 0.08377 |
| 3 | 5.45883 | 2.72942 | 1.36559 |
| 4 | 19.13858 | 9.56929 | 4.79305 |
| 5 | 22.09345 | 11.04673 | 5.52891 |
| 6 | 13.00107 | 6.50053 | 3.23738 |
| 7 | 1.02131 | 0.51066 | 0.23879 |
| 8 | 0.33043 | 0.16522 | 0.08215 |
| 9 | 0.3216 | 0.1608 | 0.07996 |
| 10 | 0.32777 | 0.16389 | 0.08106 |
| 11 | 0.33054 | 0.16527 | 0.08384 |
| 12 | 0.33318 | 0.16659 | 0.08266 |

Fig. 4C

| P=1.9 (GR3) | CI | | |
| --- | --- | --- | --- |
| Day | AL3 | AL4 | AL5 |
| 1 | 0.33804 | 0.16902 | 0.08505 |
| 2 | 0.32809 | 0.16405 | 0.08025 |
| 3 | 5.98831 | 2.99415 | 1.47384 |
| 4 | 34.33963 | 17.16981 | 8.54001 |
| 5 | 53.07792 | 26.53896 | 13.29455 |
| 6 | 53.53573 | 26.76786 | 13.37655 |
| 7 | 41.52735 | 20.76368 | 10.38977 |
| 8 | 26.8436 | 13.4218 | 6.70427 |
| 9 | 12.07141 | 6.03571 | 3.0058 |
| 10 | 0.97318 | 0.48659 | 0.23803 |
| 11 | 0.33328 | 0.16664 | 0.08241 |
| 12 | 0.33091 | 0.16545 | 0.08338 |
| 13 | 0.33267 | 0.16634 | 0.08241 |
| 14 | 0.32003 | 0.16002 | 0.07954 |
| 15 | 0.32546 | 0.16273 | 0.08147 |

Fig. 4D

| P=2.3 (GR4) | CI | | |
| --- | --- | --- | --- |
| Day | AL3 | AL4 | AL5 |
| 1 | 0.33424 | 0.16712 | 0.08323 |
| 2 | 0.33385 | 0.16693 | 0.08269 |
| 3 | 10.74832 | 5.37416 | 2.66296 |
| 4 | 58.09348 | 29.04674 | 14.55553 |
| 5 | 89.90036 | 44.95018 | 22.47855 |
| 6 | 92.0998 | 46.0499 | 22.99606 |
| 7 | 78.34024 | 39.17012 | 19.57354 |
| 8 | 63.55202 | 31.77601 | 15.89121 |
| 9 | 48.80288 | 24.40144 | 12.20961 |
| 10 | 34.08325 | 17.04162 | 8.50386 |
| 11 | 19.33582 | 9.66791 | 4.85631 |
| 12 | 4.90239 | 2.4512 | 1.23597 |
| 13 | 0.42079 | 0.21039 | 0.10582 |
| 14 | 0.32642 | 0.16321 | 0.08104 |
| 15 | 0.32997 | 0.16498 | 0.08404 |
| 16 | 0.34283 | 0.17141 | 0.08486 |
| 17 | 0.33769 | 0.16884 | 0.08445 |
| 18 | 0.32904 | 0.16452 | 0.08329 |
| 19 | 0.33269 | 0.16635 | 0.08409 |
| 20 | 0.3335 | 0.16675 | 0.08462 |

Fig. 5

| Patient Growth Ratios (P) | Max CI | | |
| --- | --- | --- | --- |
|  | AL3 | AL4 | AL5 |
| 1 | 0.33 | 0.17 | 0.08 |
| 1.4 | 4.61 | 2.3 | 1.12 |
| 1.6 | 22.09 | 11.05 | 5.53 |
| 1.9 | 53.54 | 26.77 | 13.38 |
| 2.3 | 92.1 | 46.05 | 23 |

Fig. 6A

| AL5 | Quality (Q) | | | |
| --- | --- | --- | --- | --- |
| Day | GR1 | GR2 | GR3 | GR4 |
| 1 | 1 | 1 | 1 | 1 |
| 2 | 1 | 1 | 1 | 1 |
| 3 | 1 | 0.73228 | 0.6785 | 0.37552 |
| 4 | 0.88892 | 0.20864 | 0.1171 | 0.0687 |
| 5 | 1 | 0.18087 | 0.07522 | 0.04449 |
| 6 | 1 | 0.30889 | 0.07476 | 0.04349 |
| 7 | 1 | 1 | 0.09625 | 0.05109 |
| 8 | 1 | 1 | 0.14916 | 0.06293 |
| 9 | 1 | 1 | 0.33269 | 0.0819 |
| 10 | 1 | 1 | 1 | 0.11759 |
| 11 | 1 | 1 | 1 | 0.20592 |
| 12 | 1 | 1 | 1 | 0.80908 |
| 13 | 1 | 1 | 1 | 1 |
| 14 | 1 | 1 | 1 | 1 |
| 15 | 1 | 1 | 1 | 1 |
| 16 | 1 | 1 | 1 | 1 |
| 17 | 1 | 1 | 1 | 1 |
| 18 | 1 | 1 | 1 | 1 |
| 19 | 1 | 1 | 1 | 1 |
| 20 | 1 | 1 | 1 | 1 |

Fig. 6B

| AL4 | Quality (Q) | | | |
| --- | --- | --- | --- | --- |
| Day | GR1 | GR2 | GR3 | GR4 |
| 1 | 1 | 1 | 1 | 1 |
| 2 | 1 | 1 | 1 | 1 |
| 3 | 0.99544 | 0.36638 | 0.33398 | 0.18608 |
| 4 | 0.43407 | 0.1045 | 0.05824 | 0.03443 |
| 5 | 1 | 0.09052 | 0.03768 | 0.02225 |
| 6 | 1 | 0.15383 | 0.03736 | 0.02172 |
| 7 | 1 | 1 | 0.04816 | 0.02553 |
| 8 | 1 | 1 | 0.07451 | 0.03147 |
| 9 | 1 | 1 | 0.16568 | 0.04098 |
| 10 | 1 | 1 | 1 | 0.05868 |
| 11 | 1 | 1 | 1 | 0.10343 |
| 12 | 1 | 1 | 1 | 0.40796 |
| 13 | 1 | 1 | 1 | 1 |
| 14 | 1 | 1 | 1 | 1 |
| 15 | 1 | 1 | 1 | 1 |
| 16 | 1 | 1 | 1 | 1 |
| 17 | 1 | 1 | 1 | 1 |
| 18 | 1 | 1 | 1 | 1 |
| 19 | 1 | 1 | 1 | 1 |
| 20 | 1 | 1 | 1 | 1 |

Fig. 6C

| AL3 | Quality (Q) | | | |
| --- | --- | --- | --- | --- |
| Day | GR1 | GR2 | GR3 | GR4 |
| 1 | 1 | 1 | 1 | 1 |
| 2 | 1 | 1 | 1 | 1 |
| 3 | 0.49772 | 0.18319 | 0.16699 | 0.09304 |
| 4 | 0.21703 | 0.05225 | 0.02912 | 0.01721 |
| 5 | 0.64057 | 0.04526 | 0.01884 | 0.01112 |
| 6 | 1 | 0.07692 | 0.01868 | 0.01086 |
| 7 | 1 | 0.97913 | 0.02408 | 0.01276 |
| 8 | 1 | 1 | 0.03725 | 0.01574 |
| 9 | 1 | 1 | 0.08284 | 0.02049 |
| 10 | 1 | 1 | 1 | 0.02934 |
| 11 | 1 | 1 | 1 | 0.05172 |
| 12 | 1 | 1 | 1 | 0.20398 |
| 13 | 1 | 1 | 1 | 1 |
| 14 | 1 | 1 | 1 | 1 |
| 15 | 1 | 1 | 1 | 1 |
| 16 | 1 | 1 | 1 | 1 |
| 17 | 1 | 1 | 1 | 1 |
| 18 | 1 | 1 | 1 | 1 |
| 19 | 1 | 1 | 1 | 1 |
| 20 | 1 | 1 | 1 | 1 |

Fig. 7A

| P=1.4 (GR1) | Quality (Q) | | | |
| --- | --- | --- | --- | --- |
| Day | AL3 | AL4 | AL5 | Patients |
| 1 | 1 | 1 | 1 | 278 |
| 2 | 1 | 1 | 1 | 278 |
| 3 | 0.49772 | 0.99544 | 1 | 366 |
| 4 | 0.21703 | 0.43407 | 0.88892 | 411 |
| 5 | 0.64057 | 1 | 1 | 334 |
| 6 | 1 | 1 | 1 | 284 |
| 7 | 1 | 1 | 1 | 278 |
| 8 | 1 | 1 | 1 | 278 |
| 9 | 1 | 1 | 1 | 278 |
| 10 | 1 | 1 | 1 | 278 |
| 11 | 1 | 1 | 1 | 278 |

Fig. 7B

| P=1.6 (GR2) | Quality (Q) | | | |
| --- | --- | --- | --- | --- |
| Day | AL3 | AL4 | AL5 | Patients |
| 1 | 1 | 1 | 1 | 278 |
| 2 | 1 | 1 | 1 | 278 |
| 3 | 0.18319 | 0.36638 | 0.73228 | 449 |
| 4 | 0.05225 | 0.1045 | 0.20864 | 509 |
| 5 | 0.04526 | 0.09052 | 0.18087 | 371 |
| 6 | 0.07692 | 0.15383 | 0.30889 | 298 |
| 7 | 0.97913 | 1 | 1 | 278 |
| 8 | 1 | 1 | 1 | 278 |
| 9 | 1 | 1 | 1 | 278 |
| 10 | 1 | 1 | 1 | 278 |
| 11 | 1 | 1 | 1 | 278 |

Fig. 7C

| P=1.9 (GR3) | Quality (Q) | | | |
| --- | --- | --- | --- | --- |
| Day | AL3 | AL4 | AL5 | Patients |
| 1 | 1 | 1 | 1 | 278 |
| 2 | 1 | 1 | 1 | 278 |
| 3 | 0.16699 | 0.33398 | 0.6785 | 452 |
| 4 | 0.02912 | 0.05824 | 0.1171 | 714 |
| 5 | 0.01884 | 0.03768 | 0.07522 | 438 |
| 6 | 0.01868 | 0.03736 | 0.07476 | 366 |
| 7 | 0.02408 | 0.04816 | 0.09625 | 278 |
| 8 | 0.03725 | 0.07451 | 0.14916 | 278 |
| 9 | 0.08284 | 0.16568 | 0.33269 | 278 |
| 10 | 1 | 1 | 1 | 278 |
| 11 | 1 | 1 | 1 | 278 |
| 12 | 1 | 1 | 1 | 278 |
| 13 | 1 | 1 | 1 | 278 |
| 14 | 1 | 1 | 1 | 278 |
| 15 | 1 | 1 | 1 | 278 |
